# Functional–Structural Plant Modeling Highlights How Diversity in Leaf Dimensions and Tillering Capability Could Promote the Efficiency of Wheat Cultivar Mixtures

**DOI:** 10.3389/fpls.2021.734056

**Published:** 2021-09-29

**Authors:** Emmanuelle Blanc, Pierre Barbillon, Christian Fournier, Christophe Lecarpentier, Christophe Pradal, Jérôme Enjalbert

**Affiliations:** ^1^Université Paris-Saclay, INRAE, CNRS, AgroParisTech, GQE—Le Moulon, Gif-sur-Yvette, France; ^2^Université Paris-Saclay, AgroParisTech, INRAE, UMR MIA-Paris, 75005, Paris, France; ^3^LEPSE, Univ Montpellier, INRAE, Institut Agro, Montpellier, France; ^4^UMR 1095 GDEC, INRAE, Université Clermont Auvergne, Clermont-Ferrand, France; ^5^CIRAD, UMR AGAP Institut, Montpellier, France; ^6^INRIA and LIRMM, Univ Montpellier, CNRS, Montpellier, France

**Keywords:** aerial architecture, competition for light, variety mixture, sensitivity analysis, functional traits, tillering plasticity

## Abstract

Increasing the cultivated diversity has been identified as a major leverage for the agroecological transition as it can help improve the resilience of low input cropping systems. For wheat, which is the most cultivated crop worldwide in terms of harvested area, the use of cultivar mixtures is spreading in several countries, but studies have seldom focused on establishing mixing rules based on plant architecture. Yet, the aerial architecture of plants and the overall canopy structure are critical for field performance as they greatly influence light interception, plant interactions and yield. The very high number of trait combinations in wheat mixtures makes it difficult to conduct experimentations on this issue, which is why a modeling approach appears to be an appropriate solution. In this study, we used WALTer, a functional structural plant model (FSPM), to simulate wheat cultivar mixtures and try to better understand how differences between cultivars in key traits of the aerial architecture influence mixture performance. We simulated balanced binary mixtures of cultivars differing for different critical plant traits: final height, leaf dimensions, leaf insertion angle and tillering capability. Our study highlights the impact of the leaf dimensions and the tillering capability on the performance of the simulated mixtures, which suggests that traits impacting the plants' leaf area index (LAI) have more influence on the performance of the stand than traits impacting the arrangement of the leaves. Our results show that the performance of mixtures is very variable depending on the values of the explored architectural traits. In particular, the best performances were achieved by mixing cultivars with different leaf dimensions and different tillering capability, which is in agreement with numerous studies linking the diversity of functional traits in plant communities to their productivity. However, some of the worst performances were also achieved by mixing varieties differing in their aerial architecture, which suggests that diversity is not a sufficient criterion to design efficient mixtures. Overall, these results highlight the importance of simulation-based explorations for establishing assembly rules to design efficient mixtures.

## Introduction

Increasing the cultivated diversity has been identified as a major leverage for the agroecological transition as it can help improve the resilience of low input cropping systems (Malézieux, 2012; Isbell, 2015). At the scale of the plot, diversity can be increased by mixing species or by increasing the genetic diversity within a species (varietal mixture for example). For wheat, which is the most cultivated crop worldwide in terms of harvested area (Food Agriculture Organization of the United Nations, 2018), the use of cultivar mixtures has been reported to present advantages for yield and quality as well as for diseases resistance, insect pests control, weed suppression, lodging limitation, exploitation of water and soil nutrients and yield stability (Borg et al., 2018). Moreover, the use of wheat cultivar mixtures is spreading in several countries (Faraji, 2011). In France, for example, wheat blends represented <1% of the total wheat surface in 2010 but they represented more than 11% of the total wheat surface in 2019 (FranceAgriMer, 2019). To support the spread of cultivar mixtures, assembly rules have been developed, but they're almost exclusively focused on diseases resistance (Borg et al., 2018). Studies have seldom focused on establishing mixing rules based on the plants architecture and the few existing recommendations usually advocate the association of homogeneous cultivars, but the demonstration of these guidelines seems to be lacking (Borg et al., 2018).

Yet, plant architecture and the overall canopy structure are critical for field performance, as they strongly influence light interception (Niinemets, 2010). Thus, many traits of the aerial architecture, such as height, leaf dimensions, leaf inclination and branching (i.e., tillering in cereals), have been identified for their impact on light interception. Indeed, leaf dimensions have a direct influence on the leaf area index (LAI) of the canopy, which is the most basic property affecting its light interception (Pugnaire and Valladares, 2007). Branching is also an important feature as it greatly impacts the total leaf area of the plant by changing its number of leaves (Valladares and Niinemets, 2008). On the other hand, plant height has a direct impact on the leaf area distribution in the canopy and on the overlap between neighboring leaves, which is critical for competition for light, especially in heterogeneous canopies (Givnish, 1982). As for leaf inclination, it determines the penetration of light into the canopy: the more upright the leaves are, the deeper light can penetrate into the lower layers of the canopy (Long et al., 2006). The impact of all of these traits has been considered for the definition of wheat ideotypes (i.e., ideal hypothetical plants for a given context) (Donald, 1968) and in breeding programs (Reynolds et al., 2012), but mostly in the context of single plants or pure stands. However, the impact of these architectural traits would also be of interest for the establishment of assembly rules and the definition of mixture ideotypes (Litrico and Violle, 2015). To this end, it is necessary to better understand the interactions taking place in mixtures and the role played by architectural features in these interactions.

The very high number of trait combinations in wheat mixtures makes it difficult to conduct experimentations on this issue, which is why a modeling approach appears to be an appropriate solution. In particular, functional structural plant models (FSPM, Godin and Sinoquet, 2005; Vos et al., 2010) have the advantage of being individual-based, allowing for the explicit consideration of inter-individual variations in the stand (Zhang and De Angelis, 2020). Most importantly, FSPM explicitly represent the 3D architecture of plants and its interactions with the ecophysiological processes controlling plant development. These models are thus particularly adapted to study the impact of architectural traits on the performance of heterogeneous canopies (Evers et al., 2019; Gaudio et al., 2019). If past studies using FSPM were mainly focusing on sole crops (Sarlikioti et al., 2011; Chen et al., 2014; Da Silva et al., 2014; Streit et al., 2016; Perez et al., 2018), more and more work is now addressing mixtures of different species or cultivars (Barillot et al., 2014; Munz et al., 2018; Louarn et al., 2020). For wheat cultivar mixtures, Barillot et al. (2019) have studied the impact of differences in leaf inclination during the post-anthesis period; and Vidal et al. (2018) have explored how diversity in plant height could help control rain-borne diseases. However, no FSPM study has yet focused on the impact of more than one trait on the performance of wheat cultivar mixtures.

In this study, we used an FSPM to simulate balanced binary wheat cultivar mixtures differing in four traits of their aerial architecture. Using a sensitivity analysis approach, we were able to identify the major traits impacting the performance of the simulated stands. This study improves our understanding of how differences between cultivars in key traits of the aerial architecture influence mixture performance.

## Materials and Methods

All the statistical analyses were done using the R software (R Core Team, 2018).

### WALTer: A 3D Wheat Model

WALTer (Lecarpentier et al., 2019) is an FSPM that simulates the development of the aerial architecture of winter wheat (*Triticum aestivum* L.) from sowing to maturity with a daily time step. In the model, the vegetative development of the plants (initiation, emergence, elongation and senescence of organs) follows a thermal time schedule and is based on a formalism derived from ADEL-Wheat (Fournier et al., 2003). The geometry, size, developmental kinetics and the senescence of leaves and internodes are simulated thanks to deterministic equations. However, WALTer is not a completely deterministic model, as the position of the plants, the orientation of the organs, the duration before plant emergence and the emergence of tillers are partly random.

Most importantly, WALTer simulates the competition for light between plants within the field and the resulting plasticity of tillering (i.e., the branching ability of grasses), thanks to a radiative model (CARIBU: Chelle et al., 1998). The regulation of tillering in the model is based on three simple rules. (i) Empirically fixed probabilities control the emergence of new tillers. Then, (ii) the cessation of tillering is controlled by an early neighbor perception: plants stop emitting new tillers when the surrounding Green Area Index^1^ (GAI) reaches a critical value (GAI_c_). Finally, (iii) some of the tillers that were emitted regress: a tiller regresses if the amount of photosynthetically active radiation (PAR) it intercepts per unit area falls below a threshold (PAR_t_).

Based on this formalism, WALTer produces meaningful outputs, such as the tillering dynamics of each plant (i.e., the number of axes on a plant for each day of the simulation) and the dynamics of light interception for each organ in the stand.

Since its publication in 2019 (Lecarpentier et al.), WALTer has undergone some changes aiming at improving the performances of the simulations, enhancing its realism and improving its ability to simulate mixtures of varieties, reusing several models distributed in the OpenAlea platform (Pradal et al., 2008, 2015). Thus, in the new version of WALTer, the representation of the leaves is based on the model by Fournier and Pradal (2012). The shape of the blades is based on the equations described in Dornbusch et al. (2009) and it is now possible to represent curved leaves based on the formalism described by Perez et al. (2016). The formalism for the dimensions of the leaves remains unchanged. In addition, the discretization of the sky was optimized by the use of a TURTLE sky (den Dulk, 1989) as implemented in the Python package Alinea.astk [package python]. (version 2.1.0, 2019). Furthermore, the option to consider an infinite periodic canopy, which is integrated in the radiative model (Chelle et al., 1998), was implemented in WALTer. Thus, it is no longer necessary to simulate additional plants to discard border effects. The computational cost of the simulations was further reduced by removing all dispensable (i.e., non-visible) organs from the 3D representation of plants. Moreover, a project manager, that serializes parameters, inputs, and outputs for each simulation, has been designed to distribute the computation on high-performance computing infrastructure. Modifications were also implemented to WALTer to allow the simulation of complex, heterogeneous canopies, such as varietal mixtures and populations, in which plants can differ from each other in many parameters. Finally, a fitness module has been implemented to allow the computation of the total fitness of the plot (F_tot_) for each simulation. This new output is used as a proxy of the number of kernels produced by the plot and is computed by WALTer as the sum of the fitness of all the axes in the plot. The computation of the fitness of an axis is based on the following equation:


$$\begin{array}{l} F_{axis}=\frac{PAR_{i45}}{T_{m45}} \end{array}$$

where F_axis_ is the fitness of the axis (expressed in molPAR·°Cd^−1^); PAR_i45_ is the mean daily amount of PAR intercepted by the axis during the 45 days preceding flowering (in molPAR·d^−1^); and T_m45_ is the mean temperature during the same period (in °C).

This equation is an adaptation of the photothermal quotient (Nix, 1976), which has been demonstrated to be linearly correlated to the number of grains produced in wheat (Midmore et al., 1984; Fischer, 1985; Abbate et al., 1995; Demotes-Mainard and Jeuffroy, 2001).

WALTer is available as an open source Python package on the OpenAlea platform (https://github.com/openalea/walter).

### Sensitivity Analysis

The impact of different architectural traits on the performance of wheat mixtures was evaluated through the simulation of balanced binary mixtures (mixtures of two varieties with 50% of each cultivar). To evaluate and hierarchize the influence of the different traits on the performance of the simulated mixtures, a sensitivity analysis was conducted with the variance-based Sobol method (Sobol, 1993). Because of the relatively high computational cost to run WALTer, a metamodeling approach was chosen to enable the computation of Sobol indices. The metamodel consists in a fast approximation of WALTer constructed from a limited number of model runs. We used a Gaussian process metamodel (Sacks et al., 1989; Currin et al., 1991), also known as Kriging metamodel, as in Marrel et al. (2009).

Three key outputs of the model were selected for the analyses to reflect the performance of each simulated plot. First, the mean number of ears per plant at maturity (N_ears_), which is a key component of the plot's yield, was selected. Second, the proportion of incident photosynthetically active radiation (PAR) intercepted by the stand (L_perc_), was also taken into account. For each stand, the daily L_perc_ was averaged over the whole period of use of the radiative model (from around 100 days after sowing to the maturity of the stand). Third, the total fitness of the plot (F_tot_) was used for the analyses.

To allow the comparison of the performance of a mixture with the performance of its pure components, the overyielding was also computed for each of these three outputs, using the following equation:


$$\begin{array}{l} OY_{out}=\frac{out_{mix}}{\frac{out_{pure1}+out_{pure2}}{2}} \end{array}$$

where OY_out_ is the overyielding for the considered output (N_ears_, L_perc_ or F_tot_); out_mix_ is the value of the considered output for the mixture; and out_pure1_ and out_pure2_ are the values of the considered output for each of the two components of the mixture, when cultivated in pure stands.

Five parameters associated with four key traits were considered (Table 1). For each of the five parameters, the impact of the differential between the two varieties in the mixture was considered (diff), as well as the impact of the mean value in the plot (ref). As a result, there were two input factors considered for each parameter, for a total of 10 input factors for the sensitivity analysis. The ranges of variation for all the input factors were selected to generate biologically realistic varieties with contrasted architectures. For example, the selected ranges allowed for the simulation of varieties with a final height between 40 and 160 cm (Table 1).

**Table 1:** List of parameters for the sensitivity analysis, definitions, traits impacted by the parameter, ranges of variation explored for the mean parameter value in the stand (Ref range) and for the difference of trait value between the two varieties of the mixture (Diff range), corresponding minimum and maximum values at the plant scale and units.

**Parameter**	**Description**	**Trait**	**Ref range**	**Diff range**	**Min value**	**Max value**	**Unit**
H_MS_	Final height of the main stem	Plant height	65; 135	−50; 50	40	160	cm
ϕ_B_	Insertion angle between the blade and the stem^a^	Leaf inclination	30; 50	−40; 40	10	70	degree
	Final length of the longest blade of the main stem	Leaf dimensions	16; 27	−16; +16	8	35	cm
GAI_c_	Green Area Index threshold above which the emission of tillers stops	Tillering	0.4; 0.7	−0.6; 0.6	0.1	1	–
PAR_t_	PAR threshold below which a tiller does not survive	Tillering	0.3; 0.5	−0.2; 0.2	0.2	0.6	mol·cm^−2^·°Cd^−1^

a *$L_{\max}^{B}$ also has an indirect impact on the final length of the other blades and on their width. Thus, at the scale of the cultivar, the higher the value of $L_{\max}^{B}$, the greater the blade area. See Supplementary Material for details*.

To build the Kriging metamodel, we used a set of WALTer runs following a 10 dimensions maximin latin hypercube sampling (LHS; McKay et al., 1979; Johnson et al., 1990) of 5,000 simulations generated with the DiceDesign R package (Dupuy et al., 2015) with the ranges detailed in Table 1. For each output (N_ears_, L_perc_ and F_tot_), a Kriging approximation was generated using the DiceKriging R package (Roustant et al., 2012). The sensitivity R package (Iooss et al., 2017) was then used to estimate the Sobol indices. To be able to compute the overyielding for every mixture, another set of WALTer runs was generated from a five dimensions maximin LHS of 1,000 simulations. This second LHS only includes simulations of pure stands (no trait differential in the stand) and the ranges explored for each input parameter corresponded to the minimum and maximum values detailed in Table 1. Based on this second set of simulations, a Kriging model was constructed independently for each of the three outputs (N_ears_, L_perc_ and F_tot_). Thus, the first design allowed the approximation of the behavior of mixtures while the second design allowed the approximation of the behavior of the corresponding pure components. Using the Kriging metamodels from both designs, it was possible to compute sensitivity indices for the overyielding of each output.

**Figure 1:**
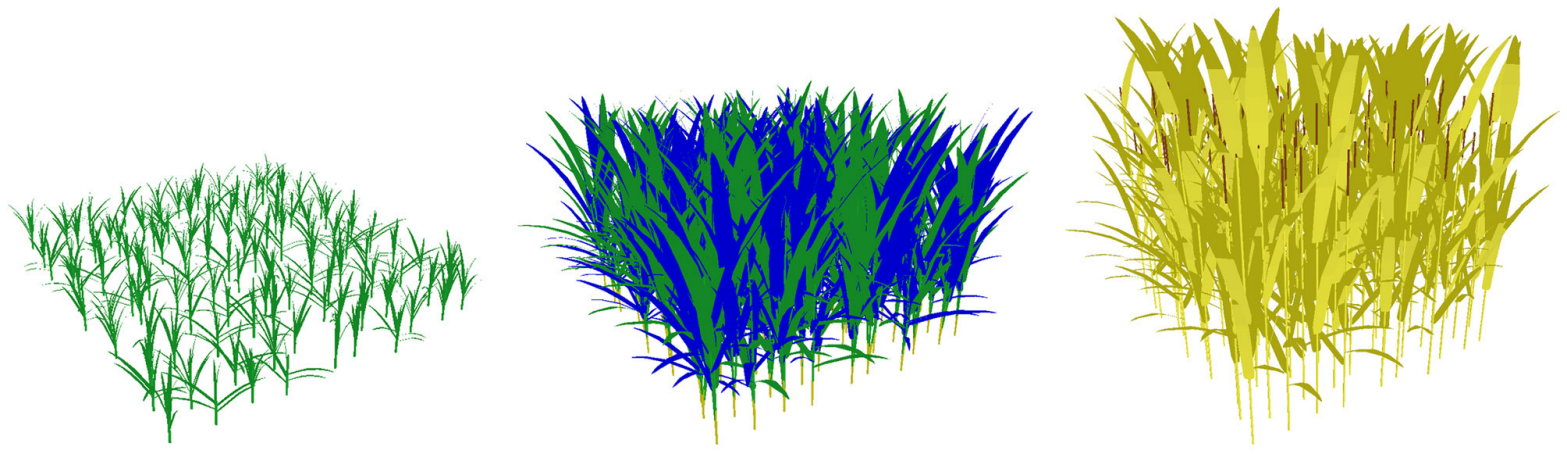
3D representation of a plot simulated with WALTer, at three different stages of development (from left to right: 100, 200 and 300 days after sowing). The simulated plot is a binary mixture composed of 110 plants sown at a density of 200 plants/m^2^. The simulation considers the plot as the pattern of an infinite periodic canopy, removing border effects. Non-senescent organs are represented in green, except for ears that are represented in brown. Regressing tillers are represented in blue and dead organs are represented in yellow.

The simulated plots were composed of 110 plants sown at a density of 200 plants/m^2^. An intermediate sowing density was chosen to allow for competition between plants while allowing plants to potentially emit and maintain several tillers. The number of plants to simulate was the object of preliminary work taking into account the impact of the stochasticity of WALTer on the model outputs (data not shown). The plants were arranged in a grid pattern with plants equidistant from each other and the different cultivars were distributed randomly in the plot (Figure 1). Apart from the input factors of the sensitivity analysis described above and in Table 1, all genotypic parameters were identical for both varieties in the mixture and had values given in Lecarpentier et al. (2019) for the cultivar Maxwell. The climatic sequence used for the simulations was obtained by averaging the climatic sequences of five French locations over 10 years (from 2007 to 2017). The five locations, listed in Supplementary Material, were selected to represent contrasted climatic conditions. For the computation of light interception, only diffuse radiations were considered, according to the standard overcast sky (Moon and Spencer, 1942). Diffuse radiation was approximated using a set of 16 light sources according to a TURTLE sky (den Dulk, 1989).

### Optimization

From the Kriging metamodels, it is possible to seek for combinations of architectural traits maximizing the performance of the stand. However, the uncertainty of the metamodels can affect the results. Therefore, we decided to enrich the initial LHS design with new runs of WALTer, based on an Efficient Global Optimization (EGO) algorithm (Jones et al., 1998). We decided to focus on the maximization of F_tot_, as this output encompasses information about both N_ears_ and L_perc_. The initial LHS design of 5,000 simulations was iteratively enriched with a new run of WALTer until 50 new runs were added. At each iteration, the parameters' values for the new run were selected based on the Expected Improvement (EI) criterion among a set of 10,000 parameter combinations sampled from a 10 dimensions latin hypercube. The EI criterion was computed with the DiceOptim R package (Roustant et al., 2012). The EI criterion is balancing the need to maximize F_tot_ with the need to reduce the uncertainty of the Kriging metamodel.

## Results

Outputs from the 5,000 initial simulations are highly variable (Supplementary Material), which indicates that at least some of the parameters varying in the LHS design have a strong impact on the performance of the simulated stands. However, some outputs are much more variable than others. Thus N_ears_ is the most variable output with values ranging from 1.0 to 4.3 ears per plant, even though 98% of the simulations resulted in N_ears_ values below 3.2 ears per plant. On the other hand, L_perc_ is much less variable, with values ranging from 0.76 to 0.96. As for F_tot_, the initial simulations gave results ranging from 0.73 to 1.5 mol·°Cd^−1^. For the OY indicators, simulations gave values between 0.40 and 1.6 for N_ears_, and values between 0.67 and 1.9 for F_tot_. Finally, the OY for L_perc_ was less variable, with values ranging from 0.97 to 1.4.

### Sensitivity Analysis

**Figure 2:**
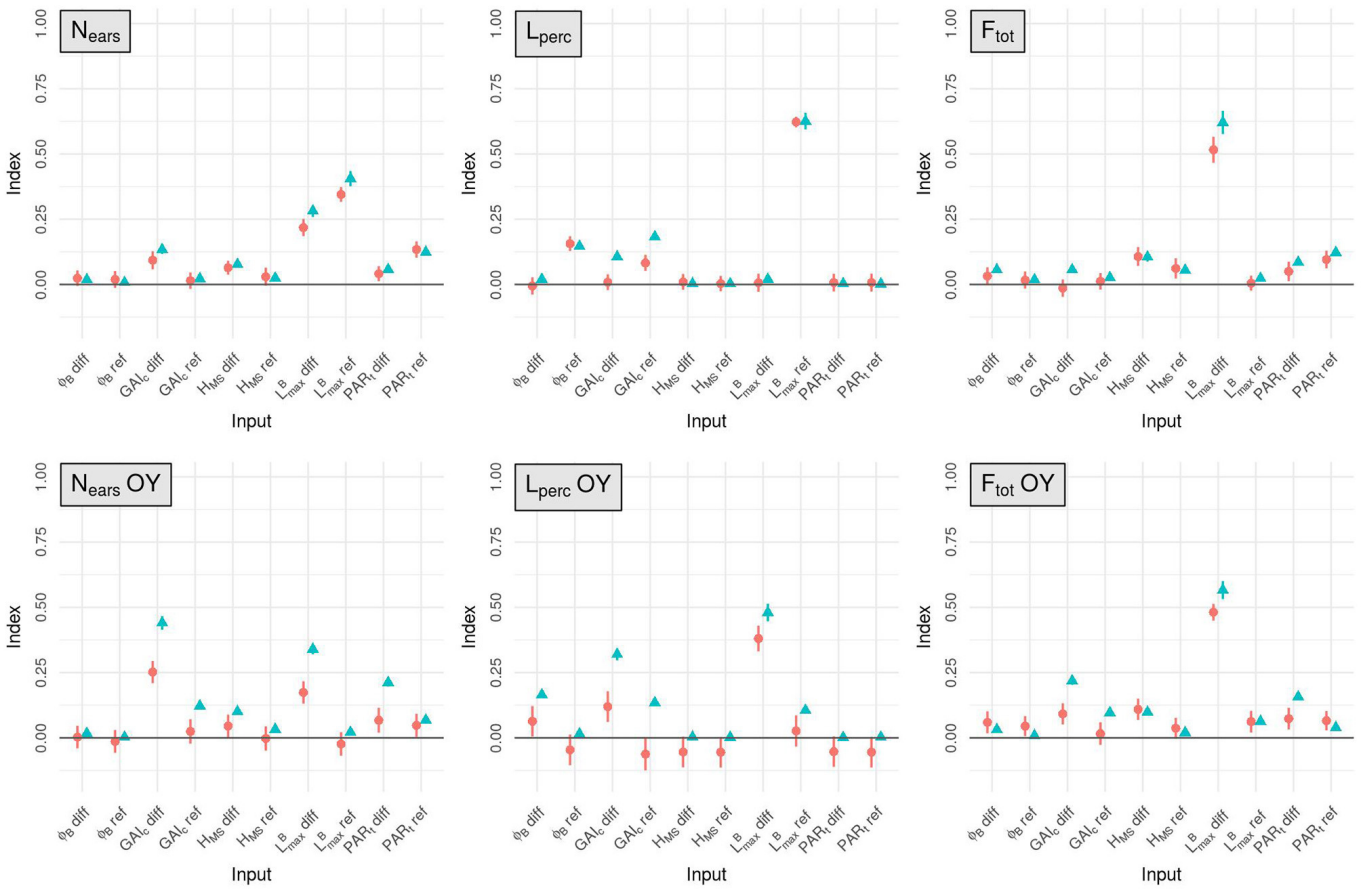
Mean estimates for the total-order (blue triangles) and first-order (red circles) Sobol indices computed for N_ears_, L_perc_, F_tot_ and the OY of these three outputs, for the 10 input factors (x-axis). The standard-deviation of each index is represented with a vertical line.

The first-order sensitivity indices (MSI) provide information on the mean influence of the different inputs on each output and the total-order indices (TSI) aggregate mean influence and influence through interactions. The most important architectural traits regarding the performance of the stands are dependent on the considered output (Figure 2).

The mean number of ears per plant (N_ears_) is mainly influenced by first-order effects. For this output the dimensions of the leaves is the most influential trait in the simulated plots as it explains more than 68% of the total variance of N_ears_ (taking into account the effect of the interactions). The mean value of $L_{\max}^{B}$ in the stand has more impact on N_ears_ than the difference of $L_{\max}^{B}$ between varieties (TSI = 0.41 and 0.28, respectively). The tillering capability of the plants in the stand also has an important impact on N_ears_: GAI_c_-diff explains around 13% of the total variance of the output and PAR_t_-ref accounts for about 12%.

For L_perc_, as for N_ears_, the input factor with the most influence is $L_{\max}^{B}$-ref. Indeed, the variations of $L_{\max}^{B}$-ref explain almost 63% of the total variance of L_perc_. GAI_c_ is also an important parameter for light interception with GAI_c_-ref accounting for about 18% of the total variance of L_perc_ and GAI_c_-diff explaining more than 10% of its total variance. However, the impact of GAI_c_ on L_perc_ is mainly explained by interaction effects. Finally, the mean leaf insertion angle in the canopy has an important impact on L_perc_ with a total-order sensitivity index of 0.15 for ϕ_B_-ref. All the other input factors have very little influence on the light interception efficiency.

The input factor with the most impact on F_tot_ is $L_{\max}^{B}$-diff (TSI = 0.62). The mean value of PAR_t_ in the mixture (PAR_t_-ref) and the height differential between varieties (H_MS_-diff) also have a lot of influence on F_tot_, as they explain 12 and 10% of the total variance of this output respectively.

For the OY outputs, the two most influential input factors are always $L_{\max}^{B}$-diff and GAI_c_-diff, regardless of the output considered. Together, these two inputs always explain more than 77% of each OY output. Interestingly, interactions between input factors have an important impact on these outputs, particularly for the OY of N_ears_ and L_perc_. Nevertheless, differences exist between the different OY outputs. For example, for the OY of N_ears_, GAI_c_-diff has more impact than $L_{\max}^{B}$-diff, whereas it is $L_{\max}^{B}$-diff that has the most influence on the OY of L_perc_ and F_tot_.

To a lesser extent, other input factors also have a substantial influence on the OY outputs. PAR_t_-diff, GAI_c_-ref and H_MS_-diff each explain more than 10% of the total variance of the OY of N_ears_. The TSI of PAR_t_-diff for the OY of N_ears_ even reaches 0.21, but this effect is mainly explained by interactions as its MSI is only around 0.067. For the OY of L_perc_, GAI_c_-ref, ϕ_B_-diff and $L_{\max}^{B}$-ref are important factors, each with a TSI >0.1. Finally, PAR_t_-diff explains almost 16% of the total variance of the OY of F_tot_.

### Optimization

**Figure 3:**
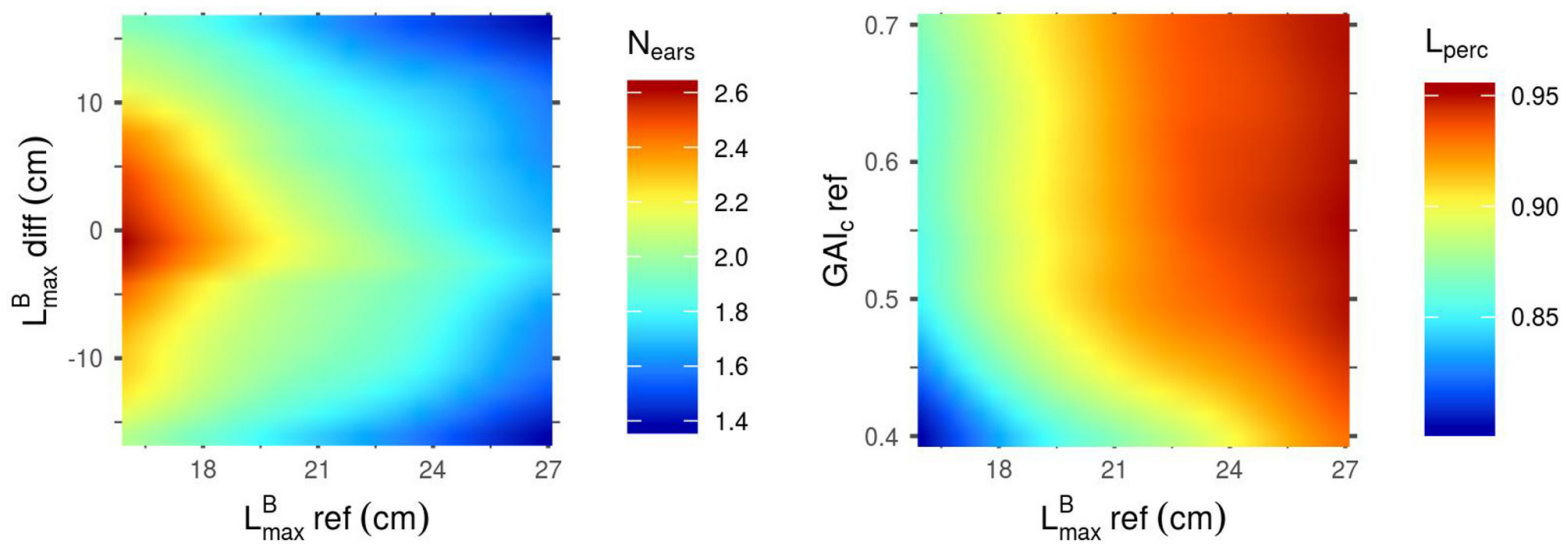
Mean outputs predicted by the Kriging metamodels as a function of their two most influential input parameters. Predictions were made with all other parameters set to their mean values. Left: N_ears_ as a function of $L_{\max}^{B}$-diff and $L_{\max}^{B}$-ref. Right: L_perc_ as a function of GAI_c_-ref and $L_{\max}^{B}$-ref.

**Figure 4:**
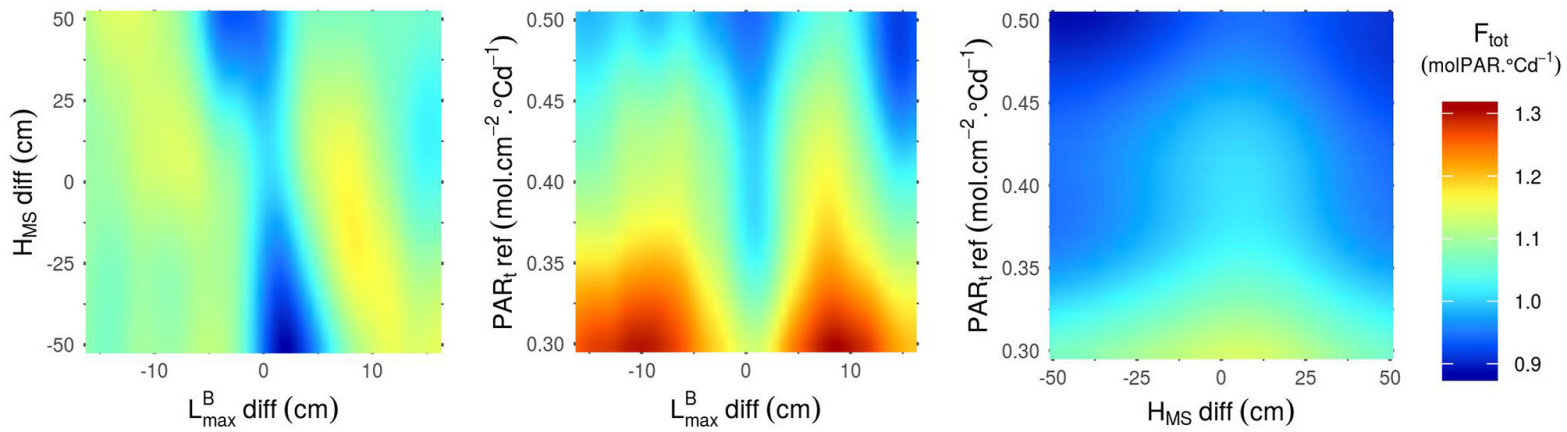
F_tot_ predicted by the Kriging metamodel as a function of the 3 parameters that have the most impact on the variance of this output ($L_{\max}^{B}$-diff, H_MS_-diff and PAR_t_-ref). Predictions were made with all other parameters set to their mean values.

**Figure 5:**
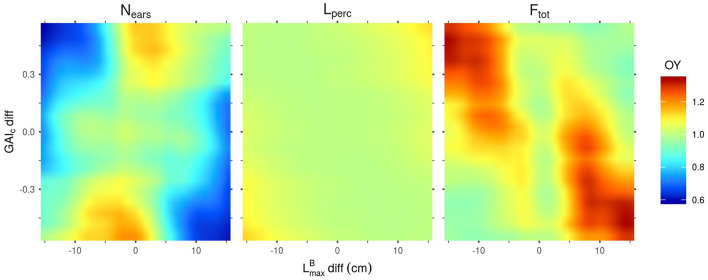
Mean overyielding (OY) predicted by the Kriging metamodels for N_ears_, L_perc_ and F_tot_ as a function of GAI_c_-diff and $L_{\max}^{B}$-diff. Predictions were made with all other parameters set to their mean values.

The sensitivity indices (Figure 2) only provide information about the relative importance of the different input parameters for the determination of the performance of the stand. Heatmaps representing the outputs of the Kriging metamodels as a function of their most influential parameters (Figures 3–**5**) give more information on how each parameter affects each output. For these figures however, it is important to keep in mind that the less influential parameters were set to the mean value of their range of variation, thus discarding most of the interaction effect that they might have with the other inputs. Another critical point when reading these heatmaps is the fact that, when the input variable is a parameter difference, it generates a plot that is symmetric on the axis diff = 0. And if the heatmap is a function of two parameters-diff, there is a central symmetry of the plot.

Figure 3 shows that the mean value of $L_{\max}^{B}$ in the stand ($L_{\max}^{B}$-ref) is negatively correlated to N_ears_, meaning that canopies with small blades tend to produce more spikes than stands with larger leaves. This heatmap also reveals that a differential of $L_{\max}^{B}$ between the cultivars of a mixture is unfavorable for the ear yield in the simulated plots. Unlike N_ears_, L_perc_ is positively correlated to $L_{\max}^{B}$-ref: larger leaves allow for a higher light interception efficiency. L_perc_ is also positively correlated to GAI_c_-ref, but mainly for values of GAI_c_-ref lower than 0.5: above this value, the impact of GAI_c_-ref on L_perc_ is very low. This means that L_perc_ is higher for stands in which plants have a low sensitivity to the early competition (high GAI_c_ values) and can thus emit many tillers.

As for F_tot_ (Figure 4), unlike the results observed for N_ears_ (Figure 3), a differential of $L_{\max}^{B}$ between the cultivars of a mixture is favorable for the performance of the stand. An absolute differential of around 10 cm between the two varieties in the mixture leads to the highest values of F_tot_. Moreover, the highest values of F_tot_ can only be reached with low values of PAR_t_-ref. Indeed, there is a clear negative correlation between PAR_t_-ref and F_tot_, which means that stands tend to be more productive when plants in the canopy do not need much light to prevent the regression of their tillers. Finally, even though the impact of H_MS_-diff on F_tot_ is low for the combinations of parameters presented in Figure 4, the results show that homogeneous plant height in the canopies lead to higher values of F_tot_ than the mixture of cultivars with a height differential. Interestingly, for mixtures of varieties with a height differential, F_tot_ is higher if the tallest variety is the one with the smallest leaves.

For the OY outputs, $L_{\max}^{B}$-diff and GAI_c_-diff are always the most influential inputs (Figure 2). However, the effect of these two inputs, when the other inputs are fixed to their mean values, are very different depending on the output considered (N_ears_, L_perc_ or F_tot_) (Figure 5). Firstly, the combinations of parameters presented in Figure 5 lead almost exclusively to OY values ≥1 for F_tot_. This means that, for these combinations of parameters, mixtures are almost always at least as performant as the mean of their pure components. Conversely, OY values for N_ears_ may be as low as 0.6 and are rarely >1. Finally, for L_perc_, the OY is mostly around 1, with very little variation in its value. For this output, the highest values of OY are obtained for the extreme values of $L_{\max}^{B}$-diff and GAI_c_-diff by mixing plants with large leaves and high values of GAI_c_ with plants with small leaves and low GAI_c_ values. For N_ears_, the highest values of OY are also obtained for the extreme values of GAI_c_-diff, but for values of $L_{\max}^{B}$-diff close to 0. For mixtures with a differential in both $L_{\max}^{B}$ and GAI_c_, the OY of N_ears_ is more important when the cultivar with the largest leaves is also the one with the highest value of GAI_c_, as is the case for the OY of L_perc_. In contrast, the OY of F_tot_ is the highest when mixing plants with large leaves and low GAI_c_ values with plants with small leaves and high GAI_c_ values. Interestingly, despite the observed differences between the combinations of parameters leading to the highest OY for N_ears_ and F_tot_, N_ears_ is positively correlated to F_tot_ in the overall dataset (data not shown). Finally, it is worth mentioning that the metamodels predict an OY of 1 for mixtures with no trait differential between cultivars (Figure 5), which means that they perform exactly like the corresponding pure stands. This is expected as all the plants in such canopies have identical parameter values. Thus, this result highlights the consistency of the metamodels' predictions.

**Figure 6:**
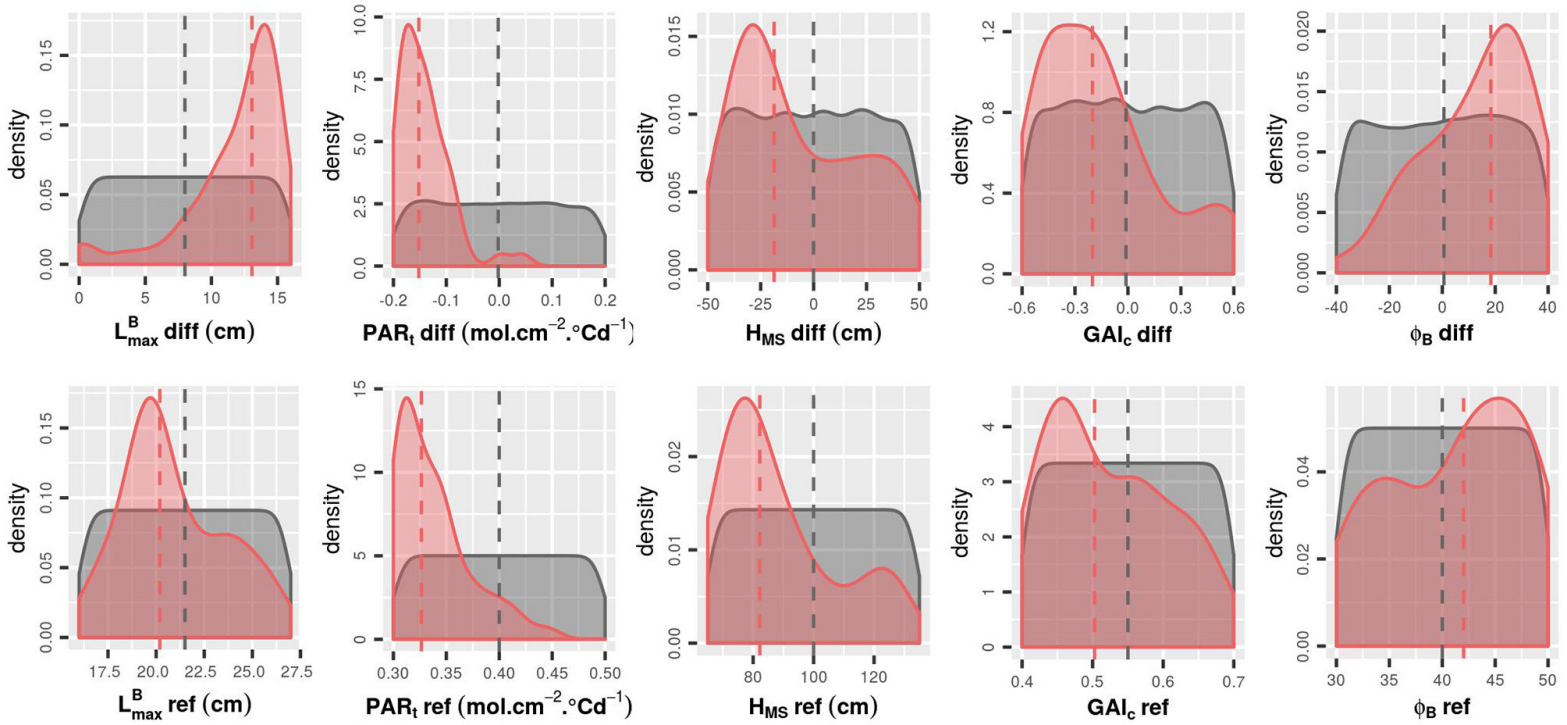
Density of each input factor for the initial LHS (gray) and for the 50 runs with the highest F_tot_ values (red). The medians for each group are represented by a dashed line. To avoid redundancy in the combinations of parameters, results are represented only with positive values of $L_{\max}^{B}$-diff.

**Figure 7:**
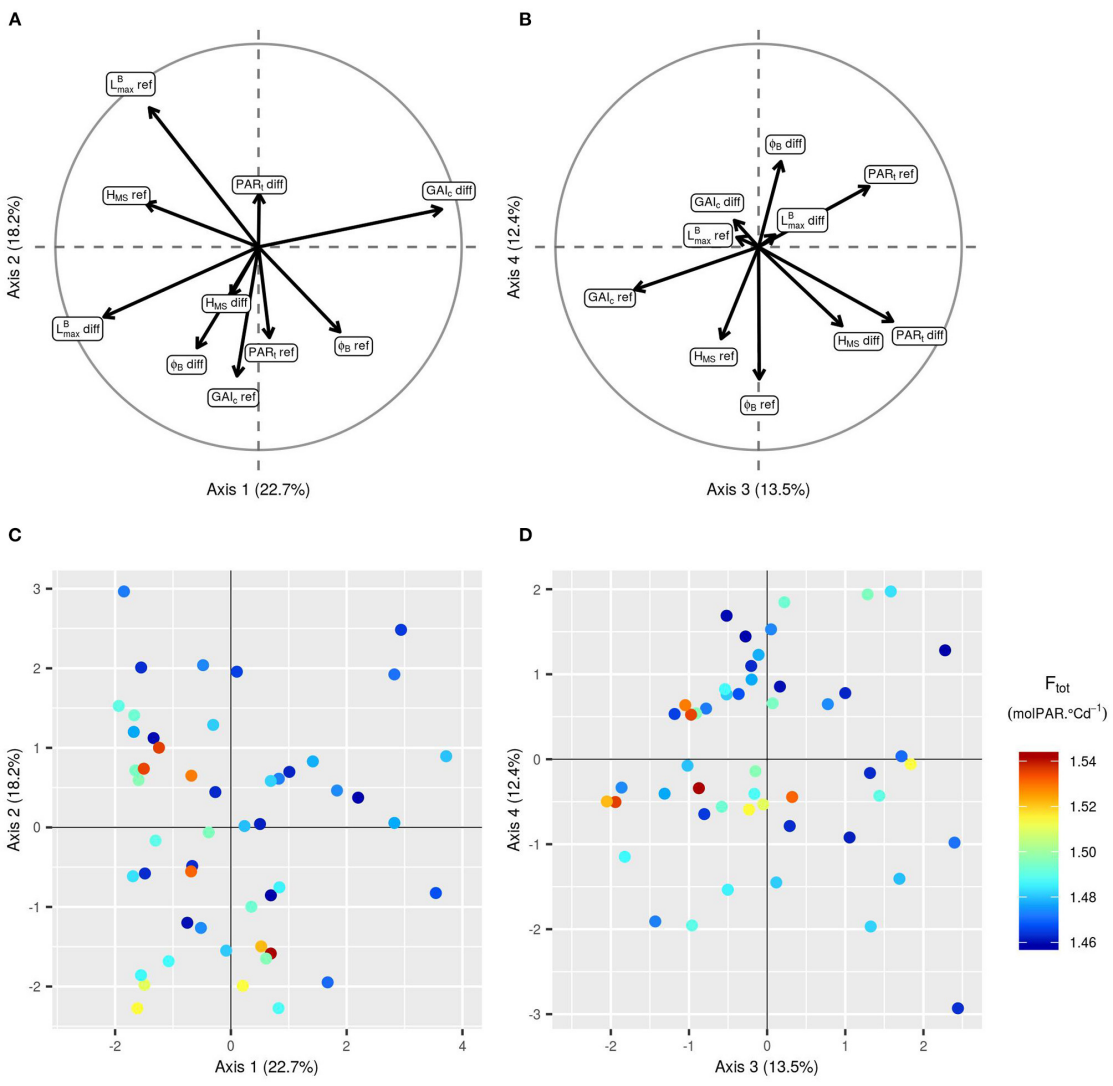
Principal components analysis (PCA) of the 10 input parameters for the 50 simulations of the initial LHS with the highest F_tot_. Projection of the 10 input parameters on the first two axes **(A)** and on the third and fourth axes of the PCA **(B)**. Projection of the 50 points on the first two axes of the PCA **(C)** and on the third and fourth axes of the PCA **(D)**. The points are colored according to the corresponding value of F_tot_. To avoid redundancy in the combinations of parameters, results are represented only with positive values of $L_{\max}^{B}$-diff.

In the initial design, different combinations of parameters can describe identical mixtures (as seen in the heatmaps). For example, considering only ϕ_B_-diff and H_MS_-diff, a stand with the minimum values of these two parameters is identical to a stand with the maximum values of the two parameters. Indeed both combinations of parameters describe a mixture of tall planophile plants with short erectophile plants. To avoid this redundancy and simplify the analysis of the results, Figures 6, 7, **9** have been represented only with positive values of $L_{\max}^{B}$-diff (all combinations are considered, but the ones with negative values of $L_{\max}^{B}$-diff have been reversed).

Focusing on the 50 runs of the initial design with the highest F_tot_ values (Figures 6, 7), it is possible to identify the trait combinations leading to the best performances. Simulations with high F_tot_ values usually combine high $L_{\max}^{B}$-diff values with low (negative) values of PAR_t_-diff. This combination of parameters describes mixtures in which the genotype with the largest leaves is the one with the lowest value of PAR_t_, i.e., the one less susceptible to tiller regression. However, there is a negative correlation between $L_{\max}^{B}$-diff and GAI_c_-diff (Figure 7), which means that it is preferable for the performance of the stand that the genotype with the largest leaves ceases tillering early in response to the neighboring competition. Furthermore, mixtures with a differential of leaf insertion angle are more likely to have high F_tot_ values if the genotype with the most erected leaves is the one with the shortest leaves. Finally, the mean values of H_MS_ and PAR_t_ in the stand should be rather low to allow for high F_tot_ values. This means that canopies of short plants with low tiller regression tend to be the most performant ones.

**Figure 8:**
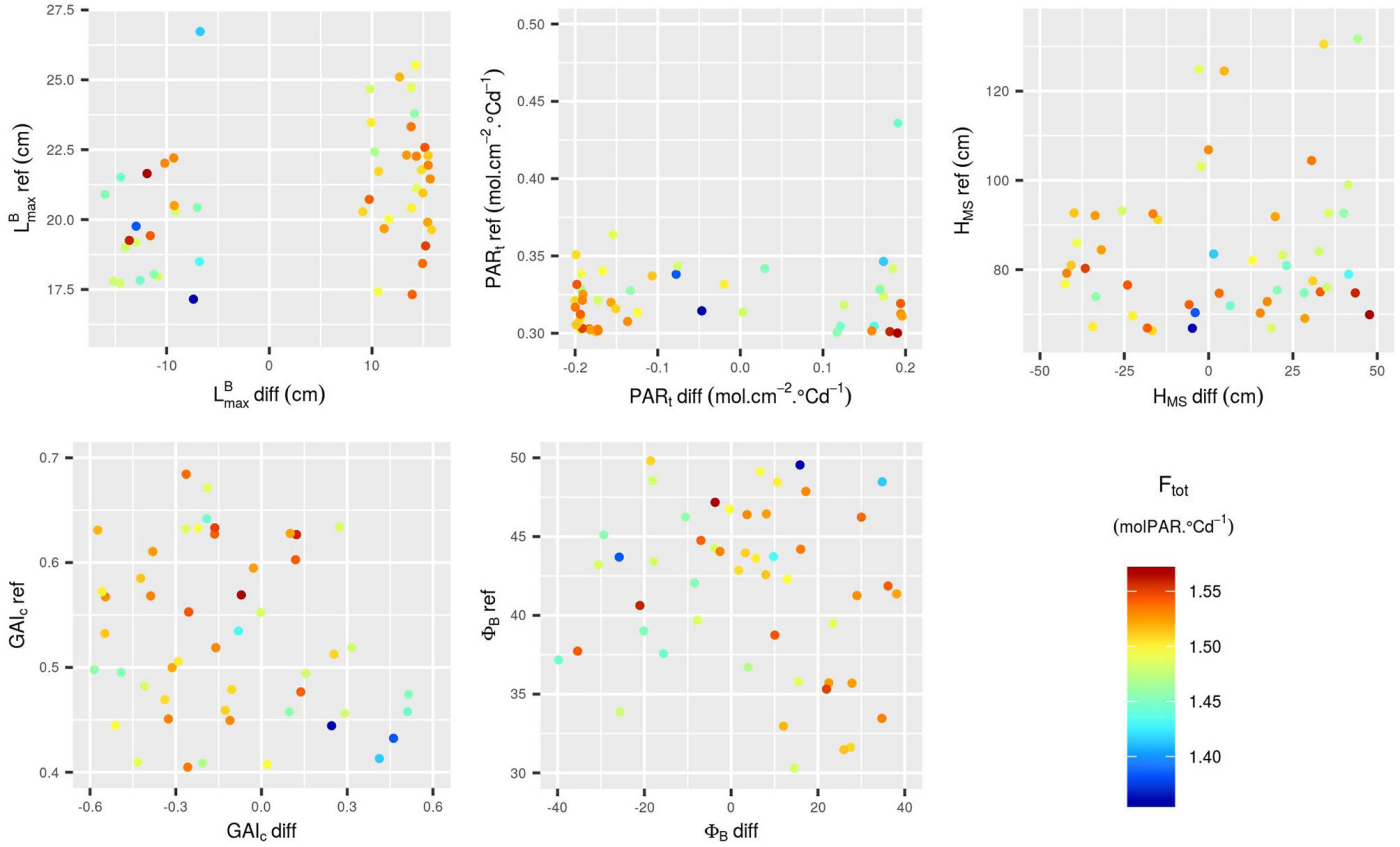
Input parameter values for the 50 points added to the design via EGO. The points are colored according to the corresponding value of F_tot_. The additional points were selected in the whole range of variation of the 10 input parameters.

**Figure 9:**
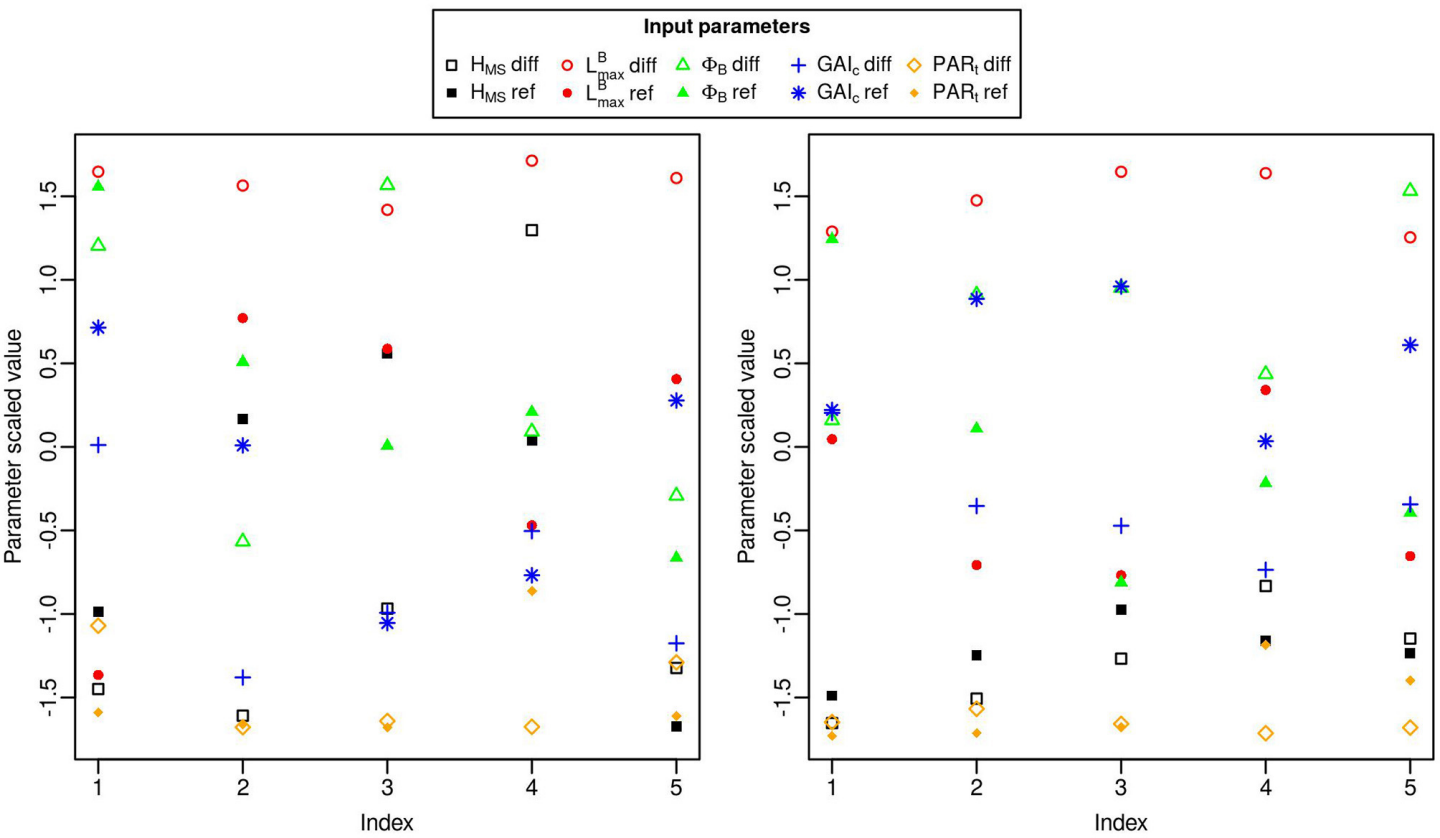
Parameter values for the five simulations with the highest F_tot_ in the initial design (left) and in the 50 simulations added by EGO (right). The x-axis is an indication of the ranking of the simulations based on the F_tot_ values. Parameter values were scaled and centered so they could all be represented on the same axis. To avoid redundancy in the combinations of parameters, results are represented only with positive values of $L_{\max}^{B}$-diff.

The addition of new runs of WALTer to the initial LHS design has confirmed the first results. In particular, for the new simulations as for the ones in the initial design, the highest values of F_tot_ are obtained for low values of PAR_t_-ref (low tiller regression) and for mixtures with a high diversity in leaf dimensions and in susceptibility to tiller regression (Figures 8, 9). When considering the initial design, the five simulations leading to the highest F_tot_ values had variable values of H_MS_-ref and H_MS_-diff (Figure 9). However, the five best-performing simulations within the 50 new runs added by EGO all had low H_MS_-ref values, meaning that high performing canopies are composed of short plants, as suggested by the results presented in Figure 6. Moreover, for the new runs of WALTer, the mixtures offering the highest F_tot_ values presented an important diversity in plant height, and the shorter genotype in the mixture was always the one with the larger leaves. This result is also in accordance with the results presented in Figure 6.

## Discussion

### FSPM as a Tool to Study Heterogeneous Canopies

This study illustrates the interest of using FSPMs to explore the impact of architectural traits on the performance of heterogeneous canopies (Evers et al., 2019; Gaudio et al., 2019; Muller and Martre, 2019; Louarn and Song, 2020; Stomph et al., 2020). Contrary to previous modeling work on wheat cultivar mixtures (Vidal et al., 2018; Barillot et al., 2019), this study focuses on multiple traits and on the entire development cycle of the plants. Thus, it provides a broader insight into the interactions taking place in wheat cultivar mixtures. Nevertheless, as for any modeling exercise (Passioura, 1996), our study presents some approximations that must be considered when interpreting the results. Indeed, even though WALTer allows for a realistic simulation of the tillering dynamics in response to competition for light (Lecarpentier et al., 2019; Blanc et al., 2021), some of the underlying hypotheses of the model should be mentioned. First of all, light is the only environmental factor considered in the model: water and nutrients (nitrogen, phosphorus, potassium, sulfur…) are not explicitly simulated by the model, even though these factors are known to impact the development of wheat. In particular, numerous studies have highlighted the important role of nitrogen in the regulation of tillering (Sparkes et al., 2006; Assuero and Tognetti, 2010; Dornbusch et al., 2011; Alzueta et al., 2012) and taking this factor into account would impact the results. Simulations are thus made with the hypothesis that resources other than light are not limiting. Moreover, plasticity was considered only for tillering, as it is the most plastic trait in response to competition for light in wheat (Lecarpentier, 2017). However, considering plasticity of other traits, and especially in leaf dimensions, could lead to different results, although it would come at a higher computational cost. Furthermore, the results are also affected by the parameterization of the model. In particular, light interception was computed using only diffuse radiations, because the simulation of more realistic sky conditions (i.e., diffuse and direct radiations) would be significantly more time-consuming. Sky conditions could have an impact on our results and additional information could be provided by the comparison of our simulations with simulations using only direct radiations (Barillot et al., 2019). Nonetheless, our preliminary studies (unpublished data) showed that there were no significant differences between the results obtained with these two conditions when simulating a simple binary mixture. Finally, even though mixtures cultivated by farmers usually combine more than two cultivars, our study focuses on the simulation of binary mixtures, as these simple mixtures allow an efficient exploration of the relations between plant architecture and stand performance.

In contrast to these limitations, the strengths inherent in the modeling approach allowed us to conduct a study that would have been impossible to achieve experimentally. Contrary to experimental studies on wheat mixtures (Borg et al., 2018; Montazeaud et al., 2020), the effect of each architectural trait on the development of the stand could be isolated from any other factor. In addition, it was possible to vary the different traits independently, without including the existing correlations between architectural traits, such as the one between leaf dimensions and leaf inclination (Ma et al., 2020), even though these biological constraints should be considered when interpreting the results. The sensitivity analysis combined with a metamodeling approach allowed us to take advantage of the complexity of an FSPM while limiting the computational time required. Thanks to the adaptive design, the uncertainty associated with the metamodeling approach was accounted for. Indeed, the addition of new runs of WALTer in areas of the parameter space that led to high values of F_tot_ improved the quality of the metamodel approximation in these areas of interest.

### Leaf Dimensions and Tillering Are Major Drivers of Mixture Performance

Our study highlights the impact of the leaf dimensions and the tillering capability on the performance of the simulated mixtures. Indeed, for all the outputs considered, those two traits were the most influential ones among the four architectural traits explored. Leaf dimensions and tillering capability have an important impact on the plant's leaf area index (LAI) whereas leaf inclination and height don't have a direct impact on the LAI but rather on the spatial distribution of the leaf area and on its orientation. Thus, our results suggest that traits impacting the LAI of plants have more influence on the performance of the stand than traits impacting the arrangement of the leaves. This result is consistent with the well-established effect of LAI on competition for light interception and productivity (Pugnaire and Valladares, 2007). Furthermore, it is in agreement with several studies using FSPMs to simulate trees (Da Silva et al., 2014; Streit et al., 2016; Perez et al., 2018), or maize intercropped with soybean (Munz et al., 2018), in which the traits related to leaf area were identified as the ones with the most influence on light interception. Finally, it is also consistent with the results of the experimental study of Montazeaud et al. (2020), in which the tillering capability was shown to have an important impact on the performance of binary wheat mixtures. The relatively smaller impact of height and leaf inclination on stand performance is congruent with the findings of Munz et al. (2018) on intercropped maize and with the study of Barillot et al. (2019) on leaf inclination in wheat cultivar mixtures. However, even though their impact on mixture performance was less important than that of tillering and leaf dimensions, plant height and leaf inclination still had an important impact on some of the performance indicators. In particular, leaf inclination had an important impact on the light interception efficiency of the simulated canopies (L_perc_) and on its OY. As for the differential of plant height between the cultivars in mixture, it was one of the most important factors influencing F_tot_ and the OY of N_ears_.

Contrary to studies on pure stands or on single plants (Sarlikioti et al., 2011; Da Silva et al., 2014; Streit et al., 2016; Perez et al., 2018), we were able to distinguish the effect of the trait differential between cultivars from the effect of the mean value of the trait in the stand, for each trait. Thus, for the leaf dimensions, the mean value in the canopy greatly impacts the light interception efficiency (L_perc_) and the number of ears produced (N_ears_), but it is mainly the differential between cultivars that influences the performance of the stand, with a major impact on all outputs except L_perc_. As for the tillering capability, the differential between cultivars and the mean value in the stand both had an important impact on mixture performance. This is in line with the experimental study conducted by Montazeaud et al. (2020), in which mixture performance was impacted by both average trait values and trait differences. In particular, they showed an important effect of both the average tillering capability and its differential on the OY of grain yield.

Interestingly, modeling studies could guide efforts to address the lack of experimental data on intraspecific mixtures. More precisely, our results suggest that it would be interesting to design experiments mixing varieties with differences in leaf dimensions and tillering capabilities. Although it is difficult to know values of these traits in wheat varieties (due to the lack of phenotypic data and the important investment required for the measurements), this type of experiment could, in turn, help with the biological validation of the models.

### Toward the Identification of Assembly Rules

Our results show that the performance of mixtures is very variable depending on the values of the explored architectural traits. In particular, the best performances were achieved by mixing cultivars with different architectures, which is in agreement with numerous studies linking the diversity of functional traits in plant communities to their productivity (Reiss and Drinkwater, 2018). However, some of the worst performances were also achieved by mixing varieties differing in their aerial architecture and the OY indicators were <1 for a significant proportion of the simulated mixtures. This suggests that diversity is not a sufficient criterion to design efficient mixtures and that random-trait assembly will not necessarily lead to better performances than pure stands, as previously stated by Louarn et al. (2020) for grassland mixtures. Overall, these results highlight the importance of establishing assembly rules to design efficient mixtures.

When focusing on the trait configurations leading to the best performances, our results converge toward a single mixture ideotype for the total fitness of the plot (F_tot_). Indeed, even if some parameters can have variable values in well performing stands, due to their low impact on F_tot_, there is no multimodality in the distribution of the parameters with high impact on F_tot_ for the best performing simulations. In particular, regarding the mean values of traits in the stand, the best-performing canopies are composed of short plants with low levels of tiller regression. Interestingly, the fact that stands with short plants perform better than stands with taller plants is in line with the assumption of a trade-off between vegetative development and reproduction. As a matter of fact, significant yield gains have been achieved by breeding for wheat cultivars with reduced height, as a shorter stature limits lodging and improves the harvest index (Gale et al., 1985). In WALTer, the negative correlation between plant height and stand performance is probably due to the formalism of tiller regression. Indeed, tiller regression depends on the amount of light intercepted per unit area, and taller tillers have a larger surface of internodes and sheaths, which makes them more likely to regress.

Regarding the differential of trait values between the two varieties in the mixture, the best-performing stands are the ones with an important diversity in both leaf dimensions and tiller regression. Our results tend to encourage the association of a very competitive genotype with a genotype with poor competitive ability, as the genotype with the largest leaves must also be the one less sensitive to tiller regression in order for the stand to perform well. This conclusion must however be qualified as the cultivar with the largest leaves should also be the shorter one and the one with the earliest cessation of tillering in response to the neighboring competition. Thus, in the best-performing plots, the most productive genotype at maturity was usually not the one that produced the most tillers. Regardless, the high F_tot_ values offered by this trait combination is explained by the very important productivity gain of the cultivar with the largest leaves, which outweighs the loss of productivity of the other cultivar in the mixture. However, it is important to specify that no plant mortality is considered in WALTer and that all individuals produce at least one ear, which is not always the case in experimental settings. In the case of the best-performing configurations that were simulated with WALTer, the cultivar with the smallest leaves had a very low productivity that might have been even lower in field conditions because of potential plant death or individuals with null productivity. Under such experimental conditions, the gain of the cultivar with the largest leaves might not have compensated for the loss in productivity of the other genotype, thus calling for caution when mixing cultivars with significant differences in their competitive ability. Moreover, the results of Montazeaud et al. (2020) showed a negative effect of diversity in tillering capability on the performance of binary mixtures of spring durum wheat sown in rows, which is in contradiction with our results. However, even if the results of our simulations are not meant to be directly translated into assembly rules, they improve our understanding of the interactions taking place in mixtures.

To further improve our understanding, it would be interesting to consider phenological traits, such as the number of days to heading or to maturity, in addition to the architectural ones. Indeed, diversity in phenology could lead to temporal complementarity in addition to the spatial complementarity offered by differences in traits of the aerial architecture. Several studies have emphasized the impact of phenological traits on the performance of wheat mixtures (Borg et al., 2018; Montazeaud et al., 2020). However, these traits were not yet considered in our study because it would require the integration of a specific module to WALTer to ensure that modifications in phenology and their implication on plant development are reliably simulated. Future work could also include the exploration of the effects of some agricultural practices. We simulated balanced mixtures (with 50% of each genotype) sown at a density of 200 plants/m^2^. However, it is expected that both sowing density and the proportions of the two cultivars in mixture should have an impact on the results (Grace and Tilman, 1990). Moreover, the sowing density has been identified, as expected, as one of the most influential inputs in WALTer (Lecarpentier et al., 2019; Blanc et al., 2021), supporting the interest in exploring variations of this parameter. FSPMs are particularly adapted to explore these agricultural practices (Evers et al., 2019; Gaudio et al., 2019). WALTer could readily be used to consider the effects of variations in both sowing density and cultivar proportion as well as their interactions with architectural traits, albeit with an increased computational cost compared to the present study.

Finally, the trait combinations identified in this study are the ones maximizing F_tot_, which is a proxy for the number of grains produced by the stand. However, cultivar mixtures can provide a wide variety of ecosystem services besides yield (Barot et al., 2017). Thus, it would be interesting to consider multiple objectives as exemplified by the study of Louarn et al. (2020) in which the stability of grassland mixtures over time was also considered in addition to yield. Considering the stability of the composition of a mixture over time could also be of interest for wheat, as this crop is often resown from year to year. The mixtures that we identified to maximize F_tot_ present significant differences in performance between their two cultivars, which would lead to a drastic evolution of their composition and possibly to a decrease in stand performance over time. Further work with WALTer would be of high interest to identify trait configurations leading to both high performance and mixture stability over time.

## Data Availability Statement

The raw data supporting the conclusions of this article will be made available by the authors, without undue reservation.

## Author Contributions

EB, JE, and PB conceived the study, designed the simulation experiments and established the statistical framework. Developments to the WALTer model were made by CF, CL, CP, and EB. EB wrote the R-scripts for the analyses and the figures with input from PB and CL. EB wrote the manuscript. All authors reviewed and approved the final manuscript.

## Funding

This work is supported by public grants overseen by the French National Research Agency (ANR) as part of the Investissement d'Avenir program, through the IDI 2017 project funded by the IDEX Paris-Saclay, ANR-11-IDEX-0003-02 and the MoBiDiv project, ANR-20-PCPA-0006.

## Conflict of Interest

The authors declare that the research was conducted in the absence of any commercial or financial relationships that could be construed as a potential conflict of interest.

## Publisher's Note

All claims expressed in this article are solely those of the authors and do not necessarily represent those of their affiliated organizations, or those of the publisher, the editors and the reviewers. Any product that may be evaluated in this article, or claim that may be made by its manufacturer, is not guaranteed or endorsed by the publisher.

## Footnotes

^1^The GAI is the ratio of photosynthetic surface of a plant to ground area.
