# Supplementary material for: Functional–Structural Plant Modeling Highlights How Diversity in Leaf Dimensions and Tillering Capability Could Promote the Efficiency of Wheat Cultivar Mixtures
Source: Front Plant Sci. 2021 Sep 29;12:734056. doi: 10.3389/fpls.2021.734056 (PMC8511389; doi:10.3389/fpls.2021.734056)
Supplement: Supplementary file 1 [file Data_Sheet_1.PDF]

Formalism for blade size

As described in Lecarpentier *et al.*, 2019, the final lengths of the blades are computed from a multi-linear function proposed in Fournier *et al.* (2003). This function depends partly on the final length of the longest blade of the axis (L_{max}^{B}). Consequently, a change in L_{max}^{B} will result in a change in the final length of all the blades of the plant emerging after the floral transition. Moreover, the maximal width of the blade is computed as a linear function of the blade's final length. Therefore, changes in the value of L_{max}^{B} also result in changes to the maximal width of the blades emerging after the floral transition. Thus, blades emerging after the floral transition are both longer and wider for plants with high L_{max}^{B} values.

Climatic sequence

The climatic sequence used for the simulations was obtained by averaging climatic sequences from Mons-en-Chaussée (49.9°N, 3.0°E) ; Marseillan-Plage (43.3°N, 3.5°E), Plomelin (47.9°N, 4.2°W), Entzheim (48.5°N, 7.6°E) and Clermont-Ferrand (45.8°N, 3.1°E).

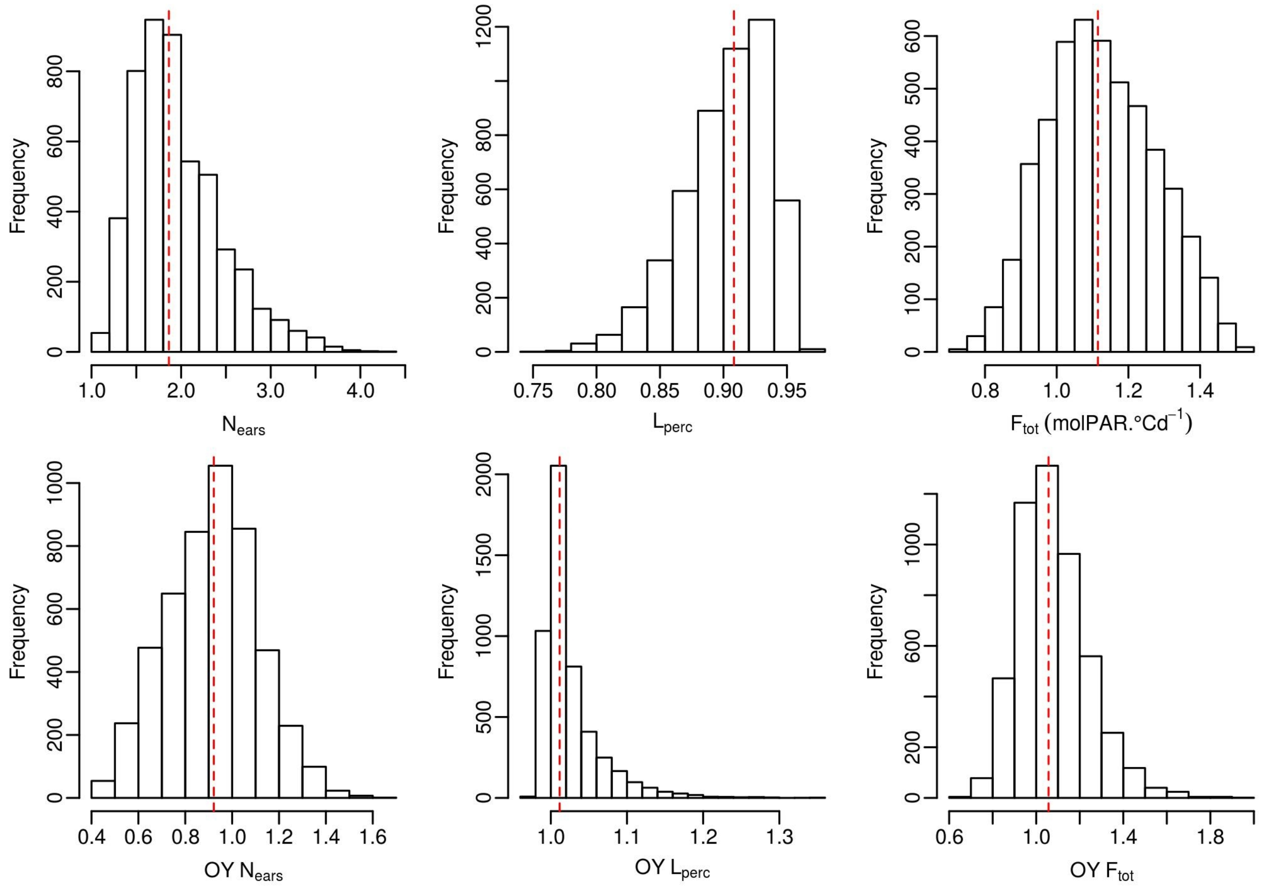

Fig. 1. Distribution of the 3 outputs (N_{ears}, L_{perc} and F_{tot}) and their OY indicators for the 5000 simulations of the initial design. The red dotted lines represent the corresponding median values.
